# Supplementary material for: Validity and reliability of the Polish version of the Self-Compassion Scale and its correlates
Source: PLoS One. 2022 May 16;17(5):e0267637. doi: 10.1371/journal.pone.0267637 (PMC9109924; doi:10.1371/journal.pone.0267637)
Supplement: S1 Appendix — (DOCX) [file pone.0267637.s001.docx]

**Appendix 1**

**Self-Compassion Scale**

**JAK ZWYKLE ZACHOWUJĘ SIĘ WOBEC SIEBIE SAMEGO W TRUDNYCH CHWILACH, KTÓRYCH DOŚWIADCZAM**

Przed udzieleniem odpowiedzi przeczytaj uważnie treść każdego zdania. W każdym punkcie wpisz (po lewej stronie), jak często zachowujesz się w określony sposób, posługując się poniższą skalą:

**Prawie nigdy Prawie zawsze**

**1 2 3 4 5**

______ 1. Jestem krytyczny/a wobec siebie i potępiam się za wady i niedociągnięcia.

______ 2. Kiedy jestem przygnębiony/a, zadręczam się i obsesyjnie skupiam na wszystkim, co jest nie tak.

______ 3. Kiedy sprawy toczą się nie po mojej myśli, postrzegam te trudności jako coś, przez co każdy w życiu przechodzi.

______ 4. Kiedy myślę o swoich niedociągnięciach, czuję się bardziej wyobcowany/a i odcięty/a od reszty świata.

______ 5. Staram się traktować siebie z miłością, gdy odczuwam emocjonalny ból.

______ 6. Kiedy zawiodę w czymś, co jest dla mnie ważne, przepełnia mnie poczucie, że do niczego się nie nadaję.

______ 7. Kiedy czuję się przegrany/a, staram się pamiętać, że na świecie jest wielu ludzi, którzy czują się podobnie.

______ 8. W naprawdę trudnych chwilach jestem wobec siebie surowy/a.

______ 9. Kiedy coś mnie martwi, staram się utrzymać emocjonalną równowagę.

______ 10. Kiedy czuję się w jakiś sposób nieudolny, staram się sobie przypomnieć, że to odczucie podziela większość ludzi.

______ 11. Jestem nietolerancyjny/a i niecierpliwy/a wobec cech mojej osobowości, które mi się nie podobają.

______ 12. Kiedy jest mi naprawdę ciężko, otaczam się troską i czułością, których potrzebuję.

______ 13. Kiedy jestem przygnębiony/a, zazwyczaj mam wrażenie, że większość ludzi jest ode mnie szczęśliwsza.

______ 14. Kiedy dzieje się coś bolesnego, staram się zachować wyważone spojrzenie na sytuację.

______ 15. Staram się postrzegać swoje niepowodzenia jako część ludzkiego życia.

______ 16. Kiedy dostrzegam w sobie cechy, które mi się nie podobają, ostro się za nie krytykuję.

______ 17. Kiedy zawiodę w czymś, co jest dla mnie ważne, staram się zachować dystans wobec tej sytuacji.

______ 18. Kiedy zmagam się z czymś bardzo trudnym, zazwyczaj wydaje mi się, że innym przychodzi to łatwiej.

______ 19. Kiedy doświadczam cierpienia, traktuję siebie z życzliwością.

______ 20. Kiedy coś mnie martwi, daję się ponieść uczuciom.

______ 21. Kiedy doświadczam cierpienia, potrafię być wobec siebie nieco chłodny/a.

______ 22. Kiedy jestem przygnębiony/a, staram się podchodzić do swoich uczuć z ciekawością i otwartością.

______ 23. Jestem wyrozumiały/a wobec swoich wad i niedoskonałości.

______ 24. Kiedy zdarza się coś bolesnego, zwykle wyolbrzymiam sytuację.

______ 25. Kiedy zawiodę w czymś, co jest dla mnie ważne, zazwyczaj czuję się samotny/a ze swoją porażką.

______ 26. Staram się z cierpliwością i zrozumieniem traktować aspekty mojej osobowości, które mi się nie podobają.

**Klucz:**

**Życzliwość**

Życzliwość (Self-Kindness): 5, 12, 19, 23, 26

Osądzanie Siebie (Self-Judgment)*: 1, 8, 11, 16, 21

**Wspólnota doświadczeń**

Wspólnota doświadczeń (Common Humanity): 3, 7, 10, 15

Izolacja (Isolation)*: 4, 13, 18, 25

**Uważność**

Uważność (Mindfulness): 9, 14, 17, 22

Nadientyfikacja (Over- -Identification)*: 2, 6, 20, 24

*Jeżeli chcemy sumować wszystkie poddskale do ogólnego wyniku self-compassion lub do trzech czynników: Życzliwość, Wspólnota doświadczeń i Uważność, itemy w saklach negatywnych (Osądzanie Siebie, Izolacja i Nadientyfikacja) należy odwrócić.

**Appendix 2 Model syntaxes**

**CFA One-factor model**

! Commands preceded by ! sign are comments that Mplus ignores.

DATA:

FILE IS C:\Users\Flakusek\Downloads\SC.dat;

! Path to and name of data file changes per study.

VARIABLE:

NAMES ARE SC1 SC2 SC3 SC4 SC5 SC6

SC7 SC8 SC9 SC10 SC11 SC12

SC13 SC14 SC15 SC16 SC17 SC18

SC19 SC20 SC21 SC22 SC23 SC24

SC25 SC26;

USEVARIABLES ARE SC1 SC2 SC3 SC4 SC5 SC6

SC7 SC8 SC9 SC10 SC11 SC12

SC13 SC14 SC15 SC16 SC17 SC18

SC19 SC20 SC21 SC22 SC23 SC24

SC25 SC26;

! Specifying that we’re treating the variables as categorical.

CATEGORICAL ARE all;

ANALYSIS:

! Requesting the weighted least squares mean- and variance-adjusted estimator

estimator = wlsmv;

MODEL:

! Specifying the latent self-compassion factor with the ‘BY’ statement

sc BY SC5* SC12 SC19 SC23 SC26 SC3

SC7 SC10 SC15 SC9 SC14 SC17

SC22 SC1 SC8 SC11 SC16 SC21

SC4 SC13 SC18 SC25 SC2 SC6

SC20 SC24

! Requesting standardized parameter estimates

OUTPUT: stdyx;

**ESEM One-factor model:**

! Commands preceded by ! sign are comments that Mplus ignores.

DATA:

FILE IS C:\Users\Flakusek\Downloads\SC.dat;

! Path to and name of data file changes per study.

VARIABLE:

NAMES ARE SC1 SC2 SC3 SC4 SC5 SC6

SC7 SC8 SC9 SC10 SC11 SC12

SC13 SC14 SC15 SC16 SC17 SC18

SC19 SC20 SC21 SC22 SC23 SC24

SC25 SC26;

USEVARIABLES ARE SC1 SC2 SC3 SC4 SC5 SC6

SC7 SC8 SC9 SC10 SC11 SC12

SC13 SC14 SC15 SC16 SC17 SC18

SC19 SC20 SC21 SC22 SC23 SC24

SC25 SC26;

! Specifying that we’re treating the variables as categorical.

CATEGORICAL ARE all;

ANALYSIS:

! Requesting the weighted least squares mean- and variance-adjusted estimator

estimator = wlsmv;

MODEL:

! Specifying the latent self-compassion factor with the ‘BY’ statement

sc BY SC5 SC12 SC19 SC23 SC26 SC3

SC7 SC10 SC15 SC9 SC14 SC17

SC22 SC1 SC8 SC11 SC16 SC21

SC4 SC13 SC18 SC25 SC2 SC6

SC20 SC24 (*1)

! Requesting standardized parameter estimates

OUTPUT: stdyx;

**CFA Two-factor model:**

! Commands preceded by ! sign are comments that Mplus ignores.

DATA:

FILE IS C:\Users\Flakusek\Downloads\SC.dat;

! Path to and name of data file changes per study.

VARIABLE:

NAMES ARE SC1 SC2 SC3 SC4 SC5 SC6

SC7 SC8 SC9 SC10 SC11 SC12

SC13 SC14 SC15 SC16 SC17 SC18

SC19 SC20 SC21 SC22 SC23 SC24

SC25 SC26;

USEVARIABLES ARE SC1 SC2 SC3 SC4 SC5 SC6

SC7 SC8 SC9 SC10 SC11 SC12

SC13 SC14 SC15 SC16 SC17 SC18

SC19 SC20 SC21 SC22 SC23 SC24

SC25 SC26;

! Specifying that we’re treating the variables as categorical.

CATEGORICAL ARE all;

ANALYSIS:

! Requesting the weighted least squares mean- and variance-adjusted estimator

estimator = wlsmv;

MODEL:

! Specifying the latent self-compassion factor with the ‘BY’ statement

pos BY SC5* SC12 SC19 SC23 SC26 SC3

SC7 SC10 SC15 SC9 SC14 SC17 SC22;

neg BY SC1* SC8 SC11 SC16 SC21

SC4 SC13 SC18 SC25 SC2 SC6

SC20 SC24;

pos@1; neg@1;

! Requesting standardized parameter estimates

OUTPUT: stdyx;

**ESEM Two-factor model:**

! Commands preceded by ! sign are comments that Mplus ignores.

DATA:

FILE IS C:\Users\Flakusek\Downloads\SC.dat;

! Path to and name of data file changes per study.

VARIABLE:

NAMES ARE SC1 SC2 SC3 SC4 SC5 SC6

SC7 SC8 SC9 SC10 SC11 SC12

SC13 SC14 SC15 SC16 SC17 SC18

SC19 SC20 SC21 SC22 SC23 SC24

SC25 SC26;

USEVARIABLES ARE SC1 SC2 SC3 SC4 SC5 SC6

SC7 SC8 SC9 SC10 SC11 SC12

SC13 SC14 SC15 SC16 SC17 SC18

SC19 SC20 SC21 SC22 SC23 SC24

SC25 SC26;

! Specifying that we’re treating the variables as categorical.

CATEGORICAL ARE all;

ANALYSIS:

! Requesting the weighted least squares mean- and variance-adjusted estimator

estimator = wlsmv;

rotation = target;

MODEL:

! Specifying the latent self-compassion factor with the ‘BY’ statement

pos BY SC5 SC12 SC19 SC23 SC26 SC3

SC7 SC10 SC15 SC9 SC14 SC17 SC22

SC1~0 SC8~0 SC11~0 SC16~0 SC21~0

SC4~0 SC13~0 SC18~0 SC25~0 SC2~0 SC6~0

SC20~0 SC24~0 (*1);

neg BY SC1 SC8 SC11 SC16 SC21

SC4 SC13 SC18 SC25 SC2 SC6

SC20 SC24

SC5~0 SC12~0 SC19~0 SC23~0 SC26~0 SC3~0

SC7~0 SC10~0 SC15~0 SC9~0 SC14~0 SC17~0 SC22~0 (*1);

! Requesting standardized parameter estimates

OUTPUT: stdyx;

**CFA Six-factor model:**

! Commands preceded by ! sign are comments that Mplus ignores.

DATA:

FILE IS C:\Users\Flakusek\Downloads\SC.dat;

! Path to and name of data file changes per study.

VARIABLE:

NAMES ARE SC1 SC2 SC3 SC4 SC5 SC6

SC7 SC8 SC9 SC10 SC11 SC12

SC13 SC14 SC15 SC16 SC17 SC18

SC19 SC20 SC21 SC22 SC23 SC24

SC25 SC26;

USEVARIABLES ARE SC1 SC2 SC3 SC4 SC5 SC6

SC7 SC8 SC9 SC10 SC11 SC12

SC13 SC14 SC15 SC16 SC17 SC18

SC19 SC20 SC21 SC22 SC23 SC24

SC25 SC26;

! Specifying that we’re treating the variables as categorical.

CATEGORICAL ARE all;

ANALYSIS:

! Requesting the weighted least squares mean- and variance-adjusted estimator

estimator = wlsmv;

MODEL:

! Specifying the latent self-compassion factor with the ‘BY’ statement

sk BY SC5* SC12 SC19 SC23 SC26;

cj BY SC3* SC7 SC10 SC15;

mi BY SC9* SC14 SC17 SC22;

sj BY SC1* SC8 SC11 SC16 SC21;

is BY SC4* SC13 SC18 SC25;

oi BY SC2* SC6 SC20 SC24;

sk@1; cj@1; mi@1; sj@1; is@1; oi@1;

! Requesting standardized parameter estimates

OUTPUT: stdyx;

**ESEM Six-factor model:**

! Commands preceded by ! sign are comments that Mplus ignores.

DATA:

FILE IS C:\Users\Flakusek\Downloads\SC.dat;

! Path to and name of data file changes per study.

VARIABLE:

NAMES ARE SC1 SC2 SC3 SC4 SC5 SC6

SC7 SC8 SC9 SC10 SC11 SC12

SC13 SC14 SC15 SC16 SC17 SC18

SC19 SC20 SC21 SC22 SC23 SC24

SC25 SC26;

USEVARIABLES ARE SC1 SC2 SC3 SC4 SC5 SC6

SC7 SC8 SC9 SC10 SC11 SC12

SC13 SC14 SC15 SC16 SC17 SC18

SC19 SC20 SC21 SC22 SC23 SC24

SC25 SC26;

! Specifying that we’re treating the variables as categorical.

CATEGORICAL ARE all;

ANALYSIS:

! Requesting the weighted least squares mean- and variance-adjusted estimator

estimator = wlsmv;

rotation = target;

MODEL:

! Specifying the latent self-compassion factor with the ‘BY’ statement

sk BY SC5 SC12 SC19 SC23 SC26

SC3~0 SC7~0 SC10~0 SC15~0

SC9~0 SC14~0 SC17~0 SC22~0

SC1~0 SC8~0 SC11~0 SC16~0 SC21~0

SC4~0 SC13~0 SC18~0 SC25~0

SC2~0 SC6~0 SC20~0 SC24~0 (*1);

cj BY SC3 SC7 SC10 SC15

SC5~0 SC12~0 SC19~0 SC23~0 SC26~0

SC9~0 SC14~0 SC17~0 SC22~0

SC1~0 SC8~0 SC11~0 SC16~0 SC21~0

SC4~0 SC13~0 SC18~0 SC25~0

SC2~0 SC6~0 SC20~0 SC24~0 (*1);

mi BY SC9 SC14 SC17 SC22

SC5~0 SC12~0 SC19~0 SC23~0 SC26~0

SC3~0 SC7~0 SC10~0 SC15~0

SC1~0 SC8~0 SC11~0 SC16~0 SC21~0

SC4~0 SC13~0 SC18~0 SC25~0

SC2~0 SC6~0 SC20~0 SC24~0 (*1);

sj BY SC1 SC8 SC11 SC16 SC21

SC5~0 SC12~0 SC19~0 SC23~0 SC26~0

SC3~0 SC7~0 SC10~0 SC15~0

SC9~0 SC14~0 SC17~0 SC22~0

SC4~0 SC13~0 SC18~0 SC25~0

SC2~0 SC6~0 SC20~0 SC24~0 (*1);

is BY SC4 SC13 SC18 SC25

SC5~0 SC12~0 SC19~0 SC23~0 SC26~0

SC3~0 SC7~0 SC10~0 SC15~0

SC9~0 SC14~0 SC17~0 SC22~0

SC1~0 SC8~0 SC11~0 SC16~0 SC21~0

SC2~0 SC6~0 SC20~0 SC24~0 (*1);

oi BY SC2 SC6 SC20 SC24

SC5~0 SC12~0 SC19~0 SC23~0 SC26~0

SC3~0 SC7~0 SC10~0 SC15~0

SC9~0 SC14~0 SC17~0 SC22~0

SC1~0 SC8~0 SC11~0 SC16~0 SC21~0

SC4~0 SC13~0 SC18~0 SC25~0 (*1);

! Requesting standardized parameter estimates

OUTPUT: stdyx;

**CFA Single-bifactor model:**

! Commands preceded by ! sign are comments that Mplus ignores.

DATA:

FILE IS C:\Users\Flakusek\Downloads\SC.dat;

! Path to and name of data file changes per study.

VARIABLE:

NAMES ARE SC1 SC2 SC3 SC4 SC5 SC6

SC7 SC8 SC9 SC10 SC11 SC12

SC13 SC14 SC15 SC16 SC17 SC18

SC19 SC20 SC21 SC22 SC23 SC24

SC25 SC26;

USEVARIABLES ARE SC1 SC2 SC3 SC4 SC5 SC6

SC7 SC8 SC9 SC10 SC11 SC12

SC13 SC14 SC15 SC16 SC17 SC18

SC19 SC20 SC21 SC22 SC23 SC24

SC25 SC26;

! Specifying that we’re treating the variables as categorical.

CATEGORICAL ARE all;

ANALYSIS:

! Requesting the weighted least squares mean- and variance-adjusted estimator

estimator = wlsmv;

MODEL:

! Specifying the latent self-compassion factor with the ‘BY’ statement

gen BY SC5* SC12 SC19 SC23 SC26

SC3 SC7 SC10 SC15

SC9 SC14 SC17 SC22

SC1 SC8 SC11 SC16 SC21

SC4 SC13 SC18 SC25

SC2 SC6 SC20 SC24;

sk BY SC5* SC12 SC19 SC23 SC26;

cj BY SC3* SC7 SC10 SC15;

mi BY SC9* SC14 SC17 SC22;

sj BY SC1* SC8 SC11 SC16 SC21;

is BY SC4* SC13 SC18 SC25;

oi BY SC2* SC6 SC20 SC24;

gen@1; sk@1;cj@1; mi@1; sj@1; is@1; oi@1;

gen WITH sk-oi@0;

sk WITH cj-oi@0;

cj WITH mi-oi@0;

mi WITH sj-oi@0;

sj WITH is-oi@0;

is WITH oi@0;

! Requesting standardized parameter estimates

OUTPUT: stdyx;

**ESEM Single-bifactor model:**

! Commands preceded by ! sign are comments that Mplus ignores.

DATA:

FILE IS C:\Users\Flakusek\Downloads\SC.dat;

! Path to and name of data file changes per study.

VARIABLE:

NAMES ARE SC1 SC2 SC3 SC4 SC5 SC6

SC7 SC8 SC9 SC10 SC11 SC12

SC13 SC14 SC15 SC16 SC17 SC18

SC19 SC20 SC21 SC22 SC23 SC24

SC25 SC26;

USEVARIABLES ARE SC1 SC2 SC3 SC4 SC5 SC6

SC7 SC8 SC9 SC10 SC11 SC12

SC13 SC14 SC15 SC16 SC17 SC18

SC19 SC20 SC21 SC22 SC23 SC24

SC25 SC26;

! Specifying that we’re treating the variables as categorical.

CATEGORICAL ARE all;

ANALYSIS:

! Requesting the weighted least squares mean- and variance-adjusted estimator

estimator = wlsmv;

rotation = target (orthogonal);

MODEL:

! Specifying the latent self-compassion factor with the ‘BY’ statement

gen BY SC5 SC12 SC19 SC23 SC26

SC3 SC7 SC10 SC15

SC9 SC14 SC17 SC22

SC1 SC8 SC11 SC16 SC21

SC4 SC13 SC18 SC25

SC2 SC6 SC20 SC24 (*1);

MODEL:

! Specifying the latent self-compassion subfactors with the ‘BY’ statement

sk BY SC5 SC12 SC19 SC23 SC26

SC3~0 SC7~0 SC10~0 SC15~0

SC9~0 SC14~0 SC17~0 SC22~0

SC1~0 SC8~0 SC11~0 SC16~0 SC21~0

SC4~0 SC13~0 SC18~0 SC25~0

SC2~0 SC6~0 SC20~0 SC24~0 (*1);

cj BY SC3 SC7 SC10 SC15

SC5~0 SC12~0 SC19~0 SC23~0 SC26~0

SC9~0 SC14~0 SC17~0 SC22~0

SC1~0 SC8~0 SC11~0 SC16~0 SC21~0

SC4~0 SC13~0 SC18~0 SC25~0

SC2~0 SC6~0 SC20~0 SC24~0 (*1);

mi BY SC9 SC14 SC17 SC22

SC5~0 SC12~0 SC19~0 SC23~0 SC26~0

SC3~0 SC7~0 SC10~0 SC15~0

SC1~0 SC8~0 SC11~0 SC16~0 SC21~0

SC4~0 SC13~0 SC18~0 SC25~0

SC2~0 SC6~0 SC20~0 SC24~0 (*1);

sj BY SC1 SC8 SC11 SC16 SC21

SC5~0 SC12~0 SC19~0 SC23~0 SC26~0

SC3~0 SC7~0 SC10~0 SC15~0

SC9~0 SC14~0 SC17~0 SC22~0

SC4~0 SC13~0 SC18~0 SC25~0

SC2~0 SC6~0 SC20~0 SC24~0 (*1);

is BY SC4 SC13 SC18 SC25

SC5~0 SC12~0 SC19~0 SC23~0 SC26~0

SC3~0 SC7~0 SC10~0 SC15~0

SC9~0 SC14~0 SC17~0 SC22~0

SC1~0 SC8~0 SC11~0 SC16~0 SC21~0

SC2~0 SC6~0 SC20~0 SC24~0 (*1);

oi BY SC2 SC6 SC20 SC24

SC5~0 SC12~0 SC19~0 SC23~0 SC26~0

SC3~0 SC7~0 SC10~0 SC15~0

SC9~0 SC14~0 SC17~0 SC22~0

SC1~0 SC8~0 SC11~0 SC16~0 SC21~0

SC4~0 SC13~0 SC18~0 SC25~0 (*1);

! Requesting standardized parameter estimates

OUTPUT: stdyx;

**CFA Two-bifactor model:**

! Commands preceded by ! sign are comments that Mplus ignores.

DATA:

FILE IS C:\Users\Flakusek\Downloads\SC.dat;

! Path to and name of data file changes per study.

VARIABLE:

NAMES ARE SC1 SC2 SC3 SC4 SC5 SC6

SC7 SC8 SC9 SC10 SC11 SC12

SC13 SC14 SC15 SC16 SC17 SC18

SC19 SC20 SC21 SC22 SC23 SC24

SC25 SC26;

USEVARIABLES ARE SC1 SC2 SC3 SC4 SC5 SC6

SC7 SC8 SC9 SC10 SC11 SC12

SC13 SC14 SC15 SC16 SC17 SC18

SC19 SC20 SC21 SC22 SC23 SC24

SC25 SC26;

! Specifying that we’re treating the variables as categorical.

CATEGORICAL ARE all;

ANALYSIS:

! Requesting the weighted least squares mean- and variance-adjusted estimator

estimator = wlsmv;

MODEL:

! Specifying the latent self-compassion factor with the ‘BY’ statement

pos BY SC5* SC12 SC19 SC23 SC26

SC3 SC7 SC10 SC15

SC9 SC14 SC17 SC22;

neg BY SC1* SC8 SC11 SC16 SC21

SC4 SC13 SC18 SC25

SC2 SC6 SC20 SC24;

sk BY SC5* SC12 SC19 SC23 SC26;

cj BY SC3* SC7 SC10 SC15;

mi BY SC9* SC14 SC17 SC22;

sj BY SC1* SC8 SC11 SC16 SC21;

is BY SC4* SC13 SC18 SC25;

oi BY SC2* SC6 SC20 SC24;

pos@1; neg@1; sk@1;cj@1; mi@1; sj@1; is@1; oi@1;

pos WITH sk-oi@0;

neg WITH sk-oi@0;

sk WITH cj-oi@0;

cj WITH mi-oi@0;

mi WITH sj-oi@0;

sj WITH is-oi@0;

is WITH oi@0;

! Requesting standardized parameter estimates

OUTPUT: stdyx;

**ESEM Two-bifactor model:**

! Commands preceded by ! sign are comments that Mplus ignores.

DATA:

FILE IS C:\Users\Flakusek\Downloads\SC.dat;

! Path to and name of data file changes per study.

VARIABLE:

NAMES ARE SC1 SC2 SC3 SC4 SC5 SC6

SC7 SC8 SC9 SC10 SC11 SC12

SC13 SC14 SC15 SC16 SC17 SC18

SC19 SC20 SC21 SC22 SC23 SC24

SC25 SC26;

USEVARIABLES ARE SC1 SC2 SC3 SC4 SC5 SC6

SC7 SC8 SC9 SC10 SC11 SC12

SC13 SC14 SC15 SC16 SC17 SC18

SC19 SC20 SC21 SC22 SC23 SC24

SC25 SC26;

! Specifying that we’re treating the variables as categorical.

CATEGORICAL ARE all;

ANALYSIS:

! Requesting the weighted least squares mean- and variance-adjusted estimator

estimator = wlsmv;

rotation = target (orthogonal);

MODEL:

! Specifying the latent self-compassion factors with the ‘BY’ statement

pos BY SC5* SC12 SC19 SC23 SC26

SC3 SC7 SC10 SC15

SC9 SC14 SC17 SC22;

neg BY SC1* SC8 SC11 SC16 SC21

SC4 SC13 SC18 SC25

SC2 SC6 SC20 SC24;

sk BY SC5 SC12 SC19 SC23 SC26

SC3~0 SC7~0 SC10~0 SC15~0

SC9~0 SC14~0 SC17~0 SC22~0

SC1~0 SC8~0 SC11~0 SC16~0 SC21~0

SC4~0 SC13~0 SC18~0 SC25~0

SC2~0 SC6~0 SC20~0 SC24~0 (*1);

cj BY SC3 SC7 SC10 SC15

SC5~0 SC12~0 SC19~0 SC23~0 SC26~0

SC9~0 SC14~0 SC17~0 SC22~0

SC1~0 SC8~0 SC11~0 SC16~0 SC21~0

SC4~0 SC13~0 SC18~0 SC25~0

SC2~0 SC6~0 SC20~0 SC24~0 (*1);

mi BY SC9 SC14 SC17 SC22

SC5~0 SC12~0 SC19~0 SC23~0 SC26~0

SC3~0 SC7~0 SC10~0 SC15~0

SC1~0 SC8~0 SC11~0 SC16~0 SC21~0

SC4~0 SC13~0 SC18~0 SC25~0

SC2~0 SC6~0 SC20~0 SC24~0 (*1);

sj BY SC1 SC8 SC11 SC16 SC21

SC5~0 SC12~0 SC19~0 SC23~0 SC26~0

SC3~0 SC7~0 SC10~0 SC15~0

SC9~0 SC14~0 SC17~0 SC22~0

SC4~0 SC13~0 SC18~0 SC25~0

SC2~0 SC6~0 SC20~0 SC24~0 (*1);

is BY SC4 SC13 SC18 SC25

SC5~0 SC12~0 SC19~0 SC23~0 SC26~0

SC3~0 SC7~0 SC10~0 SC15~0

SC9~0 SC14~0 SC17~0 SC22~0

SC1~0 SC8~0 SC11~0 SC16~0 SC21~0

SC2~0 SC6~0 SC20~0 SC24~0 (*1);

oi BY SC2 SC6 SC20 SC24

SC5~0 SC12~0 SC19~0 SC23~0 SC26~0

SC3~0 SC7~0 SC10~0 SC15~0

SC9~0 SC14~0 SC17~0 SC22~0

SC1~0 SC8~0 SC11~0 SC16~0 SC21~0

SC4~0 SC13~0 SC18~0 SC25~0 (*1);

pos@1;neg@1;

pos WITH sk-oi@0;

neg WITH sk-oi@0;

! Requesting standardized parameter estimates

OUTPUT: stdyx;
